# The Effect of Different Moderate Thermal Modification Durations on the Wood Properties of American Alder

**DOI:** 10.3390/ma15248839

**Published:** 2022-12-10

**Authors:** Honghai Liu, Zhilan Li, Xiaokai Zhang, Bin Tang, Chuan Wan, Kemin Wang

**Affiliations:** 1 College of Furnishings and Industrial Design, Nanjing Forestry University, Nanjing 210037, China; 2 Jiangsu Co-Innovation Center of Efficient Processing and Utilization of Forest Resources, Nanjing Forestry University, Nanjing 210037, China; 3 MACIO Home Co., Ltd., Chongqing 401346, China; 4 Dandong Little Ant Knowledge-Action Education Technology Co., Ltd., Dandong 158000, China

**Keywords:** thermal modification, treating time, dimensional stability, mechanical strength, wood color

## Abstract

To investigate the effect of moderate thermal modification (TM) on wood properties, American alder (*Alnus rubra*) wood was treated at 140 °C for 4 h, 8 h and 13 h, the physical and mechanical properties, dimensional stability and color changes of wood were compared and studied. The results showed that the absolute dry density of moderate-TM wood decreased significantly with time except for the 4 h treatment. Moderate TM can significantly reduce the residual stress of wood up to 90.3%. There were no significant differences in MOR and MOE between most moderate TM wood and the control group; moderate TM decreased the moisture absorption and water up-taking of wood significantly; compared to the control group, the swelling of TM wood for 13 h decreased by 24.2% and 16.0% in the tangential and radial direction, respectively, showing a significant improvement in dimensional stability. There were almost no color changes even when wood endured 140 °C and 13 h TM. The moderate TM at 140 °C for 13 h can efficiently improve wood dimensional stability and retains the natural color of wood while causing almost no damage to the wood’s mechanical strength.

## 1. Introduction

As a natural material, wood has advanced features, such as special color and grain, a high strength-to-weight ratio, easy manufacture, green and recyclable, and a special capacity for conditioning the interior temperature and relative humidity, thus it has remained consistently popular in the building, decoration and furniture manufacturing industries [1,2,3]. However, compared with other materials such as plastic and metal, wood also has some natural defects. Wood’s dimensions change with its moisture variation below the fiber saturated point (FSP); in particular, great deformations and cracks can occur when wood products are used in environments with severe temperatures or relative humidity conditions [4,5]. Furthermore, wood and wooden products also have problems with decay and insect attacks due to the unreasonable moisture in wood [6]. These defects limit their use in the field and affect the final product quality.

In order to improve wood’s properties and functions, physical and chemical modifications have been developed and applied to wood. Chemical modifications make use of certain chemical agents which react with the active groups in wood components, such as cellulose, hemicellulose and lignin, and change the chemical structure and composition of wood [7,8]. The physical and chemical characteristics of wood change due to the modification to its structure and composition [9,10,11,12,13]. Physical modifications treat wood with mechanical force or densification or using heat. Chemical modification can yield good performance, but it has potential risks to health and the environment. The densified wood also has resilience problems with changes of temperature and humidity in the use environment. Thermal modification (TM) of wood, which has a simple process without use of chemical agents, has been considered as a green method and widely used in the world. Wood is treated at high temperature using the mediums of steam, wet air, nitrogen, furnace gas or vegetable oil [2,14,15]. 

According to the definition under EU standards (CEN/TS 15679: 2007), wood thermal modification refers to changes to the cell wall composition and physical properties of wood after treatment at low oxygen temperatures above 160 °C. The quality and utilization of wood can be significantly improved by high temperatures (200–240 °C) TM [16,17]. High-temperature TM can effectively reduce the hygroscopicity and improve the dimensional stability [18,19,20,21], rot resistance and durability of wood [22]. The service life of modified wood is improved, leading to an enhancing of wood carbon fixation capacity. However, high-temperature TM wood still has poor performance in some areas, such as severe color changes, increased brittleness, and lowered density and mechanical strength [23,24,25,26]. In particular, the bending strength and screw holding force of modified wood are seriously decreased, which significantly affects subsequent wood processing. Thus, moderate-temperature (120–160 °C) TM [27] could be an alternative treatment of wood used to achieve ideal results for wood quality. A previous study showed that moderate-temperature (140 and 160 °C) TM to wood can achieve good performance in terms of mechanical strength and dimensional stability and that the comprehensive properties of wood are acceptable for further processing [28]. 

Alders are members of the birch family (Betulaceae). American alder (red alder) is the only one that reaches commercial size and abundance. It is also the most common and important of the hardwoods in the Pacific Northwest. The wood of red alder is evenly textured with a subdued grain pattern, and it is of moderate weight and hardness, qualities which are widely used in face veneer, furniture, cabinets, and woodenware [29,30]. In this study, American alder (*Alnus rubra*) wood was treated at 140 °C for 4 h, 8 h and 13 h, and the effects of heating durations on the physical properties, mechanical strength, dimensional stability and color change of wood were investigated. The objective of this study was to find a practical, moderate-temperature TM technology to improve wood dimensional stability, which would retain wood’s natural color and cause minimal damage to wood’s mechanical properties and the subsequent processing of wood in actual production. 

## 2. Materials and Methods

### 2.1. Materials

American alder (*Alnus rubra*) timbers (100 × 25 × 2500 mm, tangential × radial × longitudinal) were collected from MACIO Home Co., Ltd., Chongqing, China. The basic density and average MC of the wood were 0.37 g/cm^3^ and 10%, respectively. The timbers were sawn into samples of 100 mm (T) × 25 mm (R) × 400 mm (L), which were free of checks, decay, knots and discoloration. In total, four groups of samples were prepared, and there were four replicates in each group. One group was used as a control, while the other groups, A, B, and C, were used for the TM groups at 140 °C/13 h, 140 °C/4 h, 140 °C/8 h, respectively.

### 2.2. Equipment and Instruments

The equipment used for wood TM was a heating chamber (PR-6075, Guangdong Hongzhan Technology Co., Ltd., Hongzhan, China). The temperature and relative humidity inside the equipment can be controlled up to 160 °C and 100%, respectively. Other equipment included an oven drying chamber (DHG–9075A, Shanghai Yiheng Scientific Instrument Co., Ltd., Shanghai, China), an electronic balance (UTP-313, Shanghai Hochoice Apparatus Manufacturer Co., Ltd., Shanghai, China), a digital caliper (G101-103-101, 0.01mm, Shanghai Fuley Measuring Equipment Co., Ltd., Shanghai, China), and a universal testing machine e (WDW-30G, Jinan Tianchen Experimental Machinery Manufacturing Co., Ltd., Jinan, China).

### 2.3. Thermal Modification

Three sets of samples [100 mm (T) × 25 mm (R) × 400 mm (L)] were thermally modified by the schedule A, B and C. Each set has 4 replicates. A was a practical TM schedule of MACIO Home Co., Ltd., which was provided by the equipment supplier. B and C were the schedules used to shorten the heat treatment durations. Before TM, all samples were dried in the equipment in order to further decrease the water in the wood. The detailed stages and parameters were summarized in Table 1. All schedules included drying, treating and cooling stages, but the drying stages of schedule B and C were divided into slow and fast drying to reduce the defects resulting from fast water removal. For the drying in schedule A, temperature increased 5 °C per 1.5 h, thus it took 30 h in total to reach the aimed-for 140 °C. As the temperature increased to 140 °C, the TM process started and lasted 13, 4 and 8 h for schedule A, B and C, respectively. After that, samples were cooled in the equipment to 60 °C for 5 h. Finally, the heating and relative humidity controlling were stopped, and the door of the equipment was opened; all samples were further cooled in the equipment to room temperature for 10 h. 

### 2.4. Density in Absolute Dry Stage

Density in the absolute dry stage was determined according to the National Standard of GB/T 1933-2009. A modified timber from each group was made into samples (10 replicates) of 20 mm (R) × 20 mm (T) × 20 mm (L), and then samples were marked in the middle of each side to measure the tangential, radial and longitudinal dimensions. After that, all samples were dried in the oven at 103 ± 2 °C to a constant weight. The dimensions in the absolute dry stage were measured using a digital caliper. The density ($\rho_{0}$) in absolute dry stage was calculated by Equation (1).

(1) $$\rho_{0}=\frac{m_{0}}{V_{0}}$$

where *m*_0_ is the absolute dry mass (g) and *v*_0_ is the absolute dry volume (cm^3^).

### 2.5. Residual Stress of Wood

**Figure 1:**
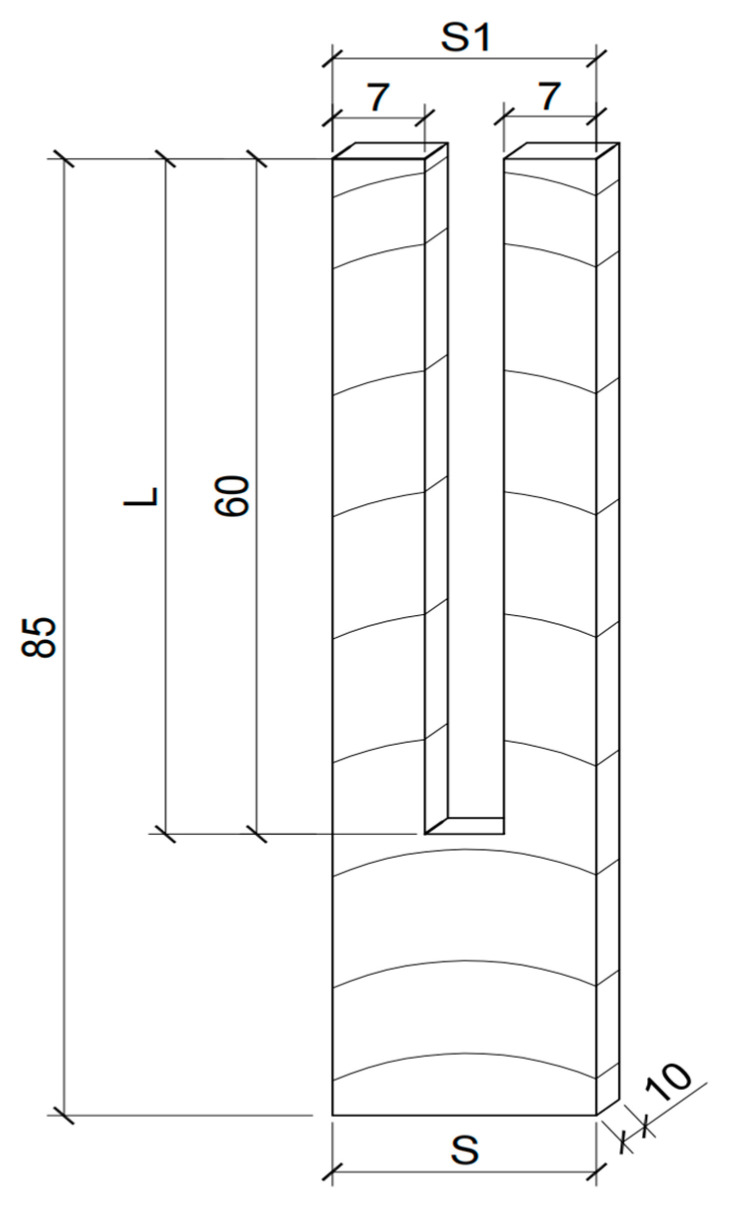
The prong samples for residual stress measurement (Unit: mm).

The prong test method was applied to measure the residual stress of the control and modified wood according to the National Standard of GB/T 6941-2012. Ten slices with the dimensions 85 mm (T) × 20 mm (R) × 10 mm (L) were sawn from the middle location of one modified timber. Each slice was cut into a prong shape using a handsaw (Figure 1). The initial dimension (*S*) in the radial direction and the length (*L*) of the prong edge were measured. After that, all slices were dried in the oven at 103 ± 2 °C for 3 h, and then cooled in a ventilated place at room temperature for 24 h. Finally, the final dimensions between two ends of the prongs (*S*_1_) were measured again.

(2) $$\;\;\;Y=\frac{S-S1}{2L}\times 100\%$$

where *Y* is the residual stress (%); *S* is the initial dimension of the slices (mm); *S*_1_ is the final dimension between two ends of the prongs after cooling (mm); and *L* is the length of prong edge (mm).

### 2.6. Mechanical Strength

The bending strength, modulus of rupture (MOR) and modulus of elasticity (MOE) of the control and modified wood were determined by the National Standard of GB/T 1936.1-2009. Samples of 20 mm (R) × 20 mm (T) × 300 mm (L) were produced from the modified wood and then conditioned at 20 ± 2 °C with 65 ± 3% relative humidity until the weight became constant. In this study, ten replicates in each group were used to measure the MOR and MOE using a bending test machine (WDW-30G, Jinan Tianchen Experimental Machinery Manufacturing Co., Ltd., Jinan, China).

### 2.7. Moisture Adsorption and Water Uptake

Moisture adsorption and water uptake were measured by GB/T-1931-2009. Absolute dried samples of the control and modified wood with dimension of 20 mm (R) × 20 mm (T) × 20 mm (L) were used for these two tests. For moisture adsorption, the mass and dimensions of the absolute dried samples in the tangential, radial, and longitudinal directions were firstly measured, then they were conditioned in a chamber at 20 ± 2 °C with 65 ± 3% relatively humidity (RH) until their weight became constant. During the conditioning process, the mass and dimensions in three directions were measured by an electronic balance and a digital caliper. The moisture adsorption capacity was indicated by equilibrium moisture content (EMC). For the water uptake test, another 4 groups of samples (10 replicates in each group) were placed in a plastic box filled with distilled water; similar to the moisture adsorption test, the mass and dimensions measurements were performed during the water uptake process. When the weight became constant, all samples were measured again for mass and dimensions after cleaning the water from the sample surfaces. The water uptake capacity was presented as EMCW. The EMC and EMCW were calculated using Equations (3) and (4).

(3) $$\mathrm{EMC}=\frac{\left(m_{e}-m_{0}\right)}{m_{0}}\times 100\%$$

(4) $$\mathrm{EMCW}=\frac{\left(m_{w}-m_{0}\right)}{m_{0}}\times 100\%$$

where *m_e_* is the weight after conditioning (g), *m*_0_ is the absolute dry weight (g), and *m_w_* is the weight after water uptake (g).

### 2.8. Dimensional Stability

Dimensional stability is evaluated by swelling in the tangential and radial directions of the control and modified samples after equalization in the conditioning chamber. The swelling after chamber conditioning (*S*) was calculated according to Equation (5):

(5) $$S=\frac{l-l_{0}}{l_{0}}\times 100\%$$

where *l* is the dimensions in the tangential or radial direction after equalization in the conditioning chamber and *l*_0_ is the absolute dried dimension in the tangential or radial directions.

### 2.9. Colour Measurements

Wood color was measured at the same five sites on the timber surface before and after TM. A digital image was obtained by a camera, and then the color data of L*, a* and b* at the measured sites were collected by the Photoshop software [31,32] as the mean value of the five images. The CIE L*, a*, and b* space coordinates were determined, and the color changes, ∆E*, were calculated using Equation (6):

(6) $$∆E^{*}=\sqrt[{2}]{\left(∆a^{2}+∆b^{2}+∆L^{2}\right)}$$

where ∆L*, ∆a*, and ∆b* are the changes to the lightness, green–red and blue–yellow chromatic coordinates before and after TM, respectively.

### 2.10. Statistical Analysis

Statistical Product Service Solutions (SPSS) was used to conduct an analysis of variance (ANOVA) in order to evaluate the effect of TM on wood, and significant differences between the mean values of the control and treated samples were determined using Duncan’s multiple range tests (*p* < 0.05).

## 3. Results and Discussion

### 3.1. Density in the Absolute Dry Stage

**Figure 2:**
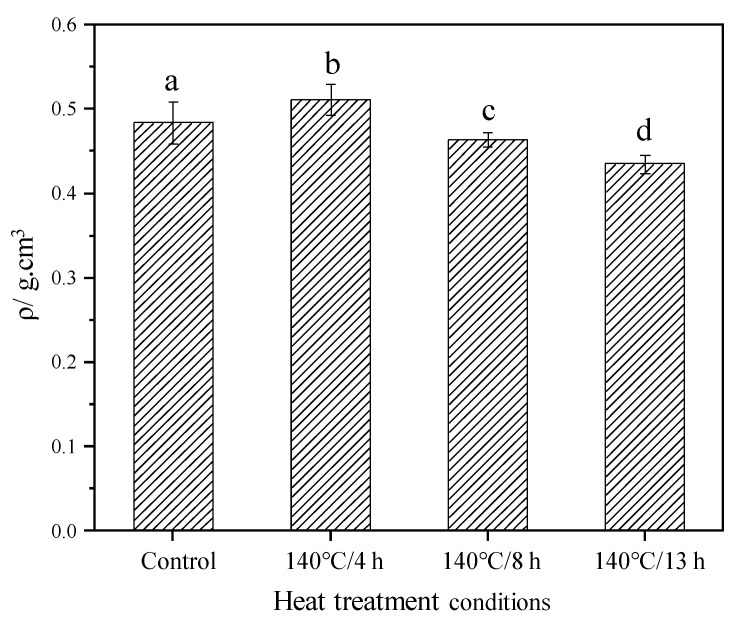
Absolute dry density of the control and modified wood. Bars with different letters (a, b, c and d) indicate significant differences (*p* < 0.05) according to Duncan’s multiple range tests.

The density of the control and modified wood in the absolute dry stage is presented in Figure 2. Compared to the control group, the density of samples decreased by 4.1% and 10.1%, respectively, after 8 and 13 h TM, indicating a significant decrease with the severity of treatment conditions. Meanwhile, significant differences were observed between all groups by analysis of variance (*p* < 0.05), indicating heat treatment duration has a significant effect on density. However, the behavior of the modified samples for 4 h indicated an opposite trend in which the density was higher than the control group. The decreased density is mainly attributed to the mass loss of samples due to the degradation of hemicellulose, the reaction of a small amount of cellulose and lignin, and the volatilization of the extractives [33]. Furthermore, small fractures which occurred due to TM may increase the volume of wood, this also leading to a decrease in density [28]. However, for the increased density of 4 h TM samples, one reason could be that the samples in this group had an inherent higher density before TM, and that the short duration resulted in less mass loss and volume change.

### 3.2. Residual Stress of Wood

**Figure 3:**
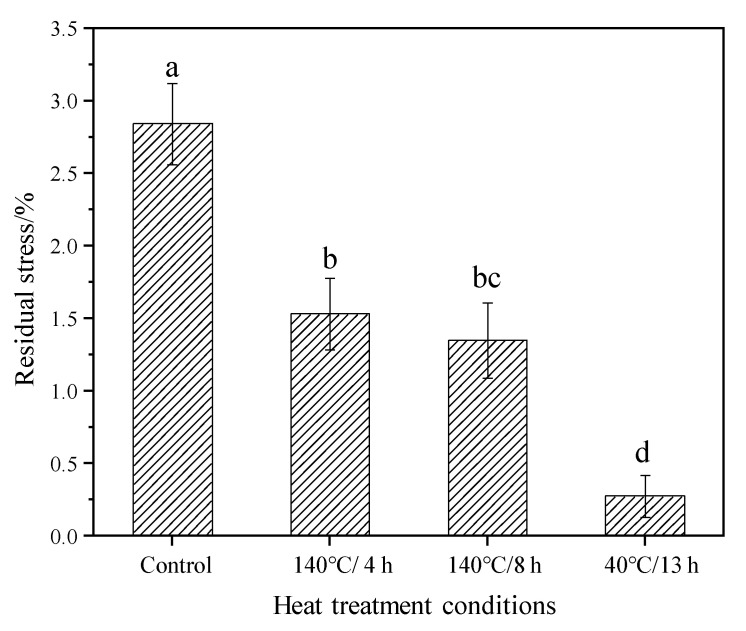
The residual stress of the control and modified wood. Bars with different letters (a, b, bc and d) indicate significant differences (*p* < 0.05) according to Duncan’s multiple range tests.

The residual stresses of the control and modified samples are illustrated in Figure 3. The stress value of the control group is about 2.8%, which meets the level-2 standard (GB/T 6941-2012). However, the residual stress after TM decreased significantly compared to the control group, with a decrease of 46% (4 h), 52.5% (8 h) and 90.3% (13 h), respectively. The residual stress values of TM wood meet the level-1 standard (GB/T 6941-2012). These results indicate that TM can significantly reduce the residual stress in wood, and the reduction degree depends on the treatment duration. The temperature and moisture affect the strain changes of wood [34,35]. The stress relaxation occurs as the temperature rises to a certain degree, and it increases sharply with the increase of temperature. Furthermore, a high temperature steam also can release the stress inside the wood [36]. The relaxation and releasing of stress are mainly caused by the changes of wood components due to TM, namely hemicellulose chain breaking, lignin molecular cracking, cellulose crystallinity increase and a bridging between the main components of the cell wall [37].

### 3.3. Mechanical Strength

**Figure 4:**
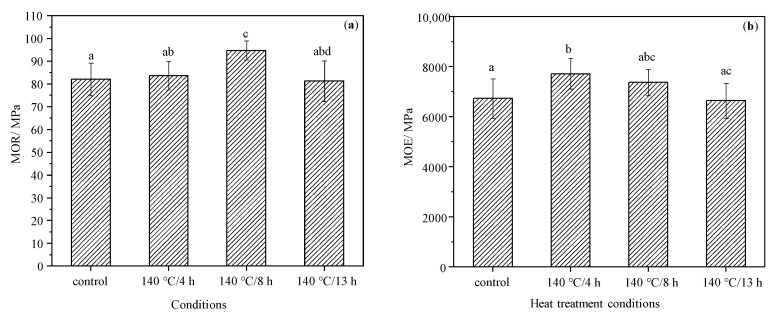
The MOR (**a**) and MOE (**b**) of the control and modified wood. Bars with different letters (left: a, ab, c and abd, and right: a, b, abc and ac) indicate significant differences (*p* < 0.05) according to Duncan’s multiple range tests.

The MOR and MOE of all samples are demonstrated in Figure 4. The MOR of 8 h TM wood increased by 14.5% compared to the control group, indicating a significant difference from other groups, while the MOR of 4 h and 13 h TM wood did not present significant differences from the control (*p* < 0.05). However, the MOE of 4 h TM wood has significant differences to the control and 13 h TM groups; the MOE of modified wood in the 8 h and 13 h groups does not show significate differences from the control group. The ANOVA shows that there was no significant difference in MOR or MOE between most modified wood and control wood, indicating that treating durations have no significant effect on the MOR and MOE of wood undergoing 140 °C TM. Hemicellulose degradation during the TM process plays an important role in reducing wood strength [38]. The increased MOR of 8 h TM and MOE of 4 TM wood could be due to less degradation of wood components, in particular hemicellulose, and to the increasing of crystallinity of the cellulose [39,40].

### 3.4. Moisture Adsorption and Water Uptake

**Figure 5:**
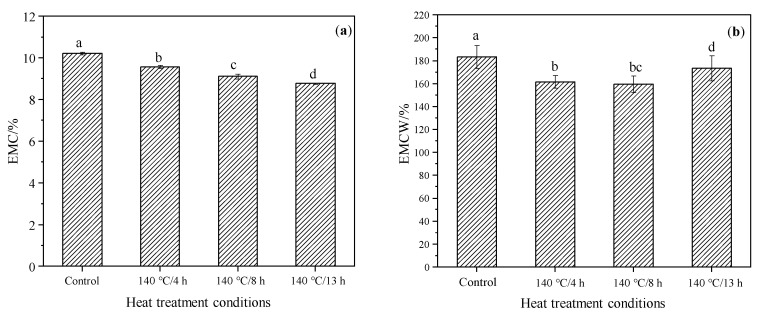
The EMC (**a**) and EMCW (**b**) of the control and modified wood. Bars with different letters (left: a, b, c and d, and right: a, b, bc and d) indicate significant differences (*p* < 0.05) according to Duncan’s multiple range tests.

The capacity for moisture adsorption and water uptake of the control and modified samples are demonstrated by EMC and EMCW, which are illustrated in Figure 5a,b, respectively. The EMC of the control, 4 h, 8 h and 13 h TM groups are 10.2, 9.6, 9.1 and 8.8%, respectively. The capacity of moisture adsorption decreased by 6.4, 10.8 and 14.1%, respectively, in contrast to the control group. Analysis of variance showed that there were significant differences between the control and the modified wood, indicating that TM can significantly reduce the moisture absorption of wood and that TM duration has a significant impact on the moisture absorption of wood. The EMCW of TM samples also decreased significantly compared to the control group (*p* < 0.05) but did not present a noticeable decreasing trend with treatment duration as was the case with EMC. Meanwhile, there were no significant differences in EMCW between the 4 h- and 8 h-modified groups. These results demonstrate that TM durations have less impact on the capacity of water uptake than that of moisture adsorption. The TM results in the degradation of wood cell wall components, leading to a reduction of the hydrophilic hydroxyl group and carbonyl group [41,42,43,44]. These are contributable, obviously, to decreasing the moisture adsorption capacity, but they are not noticeable for the reduction of water uptake of wood.

### 3.5. Dimensional Stability

**Figure 6:**
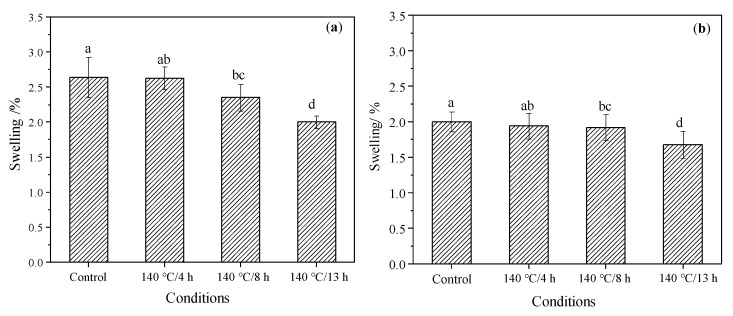
The swelling in the (**a**) tangential, and (**b**) radial direction of the control and modified wood after equalization in the conditioning chamber. Bars with different letters (left: a, ab, bc and d, and right: a, ab, bc and d) indicate significant differences (*p* < 0.05) according to Duncan’s multiple range tests.

The swelling in the tangential and radial direction of the control and modified samples after reaching equalization in the conditioning chamber are presented in Figure 6a,b, respectively. The effects of 4 h TM on swelling are not noticeable in both tangential and radial direction, but the swelling after 13 h TM decreased by 24.2% and 16.0% in the tangential and radial direction, respectively, in contrast to the control group, indicating an apparent reduction. Analysis of variance also showed that only the 13 h TM had a significant impact on the swelling in both directions (*p* < 0.05).

### 3.6. Colour Changes

**Figure 7:**
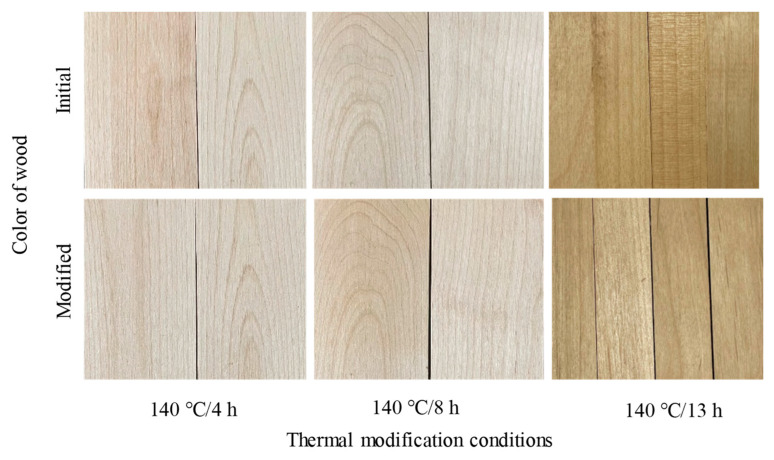
Visual comparison of control and heat-modified wood showing color.

Table 2 summarized the color data of samples prior to and after TM; meanwhile, the photos of the samples in the initial and modified stages were shown in Figure 7. The values of ΔL* are all negative after TM, indicating that the wood color darkened. Furthermore, the positive values of Δa* and Δb* demonstrate that the wood color turns a little more red and yellow after TM. The total color change of ΔE* became great when the TM duration was longer than 8 h, but there were almost no differences between the ΔE* of the 8 and 13 h modified groups. A previous study verified that color changes can be seen clearly by the naked eye only when the value of ΔE* is larger than 3 [45]. In this study, the maximum ΔE* for 8 and 13 h modified samples were a little higher than the limit, but the color changes of modified samples are very hard to distinguish with the naked eye (Figure 7). ANOVA combined with Duncan’s multiple range tests showed that, compared with a 4 h TM duration, significant changes to Δa*, Δb*, and ΔE* were observed when the TM durations exceed 8 h, while there were no significant differences between 8 h and 13 h. But, for ΔL*, only the 13 h TM duration had a significant difference. Wood color changes were caused by the chemical composition and structural changes of wood components. This is mainly related to the glucose content and the color-degradation products of hemicellulose [46,47]. Color changes were also affected by other factors [48], such as the lignin and extractives content in wood [49,50]. Thus, it can be concluded that even in the case of wood exposed to 13 h TM at 140 °C, severe changes to the composition, structure, and extractives of wood did not occur, because wood color changed only slightly.

## 4. Conclusions

American alder *(Alnus rubra*) wood was thermally modified under moderate conditions at 140 °C for 4 h, 8 h and 13 h, and the effect of the treatment duration on the properties of wood, such as absolute dry density, mechanical strength, capacity for moisture adsorption and water uptake, dimensional stability, and color changes were investigated. The results are summarized as follows: the absolute dry density of TM wood decreased significantly with treatment duration except for the 4 h TM group; the moderate TM decreased wood residual stress by 90.3% after 13 h of treatment; the moderate TM at 140 °C had almost no effect on the mechanical strength of the MOR and MOE of wood; the capacity for moisture absorption and water uptake of wood were significantly improved after moderate TM, but the effects of treatment duration on moisture absorption and water uptake were different; in contrast to the control group, the swelling of 13 h TM wood decreased by 24.2% and 16% in the tangential and radial direction, respectively, indicating that long-duration moderate TM can improve wood dimensional stability efficiently; almost no color variation was observed even when wood was TM at 140 °C for 13 h. The moderate TM technology is an effective method to improve wood dimensional stability and retain wood color, but it has less effect on the mechanical properties of wood.

## Figures and Tables

**Table 1:** Experimental process and parameters for schedule A, B and C.

Schedules	Stages	Initial T. (°C)	Aimed T. (°C)	Relative Humidity (%)	Time (h)
A	Drying	40	140	65	30
	Treating	140	140	100	13
	Cooling	140	60	100	5
B C	Slow drying	40	80	75	4
	Fast drying	80	100	30	2
	Treating (B)	100	140	100	4
	Treating (C)	100	140	100	8
	Cooling	140	60	100	5

**Table 2:** Color parameters of wood before and after heat treatment.

Treating Conditions		ΔL*	Δa*	Δb*	ΔE*
Temperature/°C	Time/h				
140	4	−1.25 a	0.08 a	0.67 a	1.65 a
140	8	−0.38 a	1.06 b	2.88 b	3.59 b
140	13	−2.91 b	0.63 b	2.38 b	3.53 b

Means followed by different letters indicate significant differences (*p* < 0.05) according to Duncan’s multiple range tests.

## Data Availability

Not applicable.
